# Sustainable Synthesis of Electroplating Sludge-Derived MOFs for CO_2_ Adsorption and Antibacterial Applications

**DOI:** 10.1021/acsomega.6c03377

**Published:** 2026-09-28

**Authors:** Jia-Yin Lin, Si-Han Yu, Yun-Ju Lai, Chih-Ying Wang, Nai-Wen Tsao, Bussaba Pinchaipat

**Affiliations:** † Graduate Program in Semiconductor and Green Technology, Academy of Circular Economy, 34916 National Chung Hsing University, Taichung 402, Taiwan; ‡ Graduate Program in Industrial and Smart Technology, Academy of Circular Economy, National Chung Hsing University, Taichung 402, Taiwan; § Innovation and Development Center of Sustainable Agriculture, National Chung Hsing University, Taichung 402, Taiwan; ∥ Department of Wood-Based Materials and Design, National Chiayi University, Chiayi 600, Taiwan; ⊥ Department of Basic Science and Physical Education, 113271 Kasetsart University, Bangkok 10900, Thailand

## Abstract

This study presents a sustainable strategy for converting electroplating sludge (EPS) into functional metal–organic frameworks (MOFs) for environmental applications. Two EPS-derived MOFs, namely, EPS-MOF-D (Cu–BDC type) and EPS-MOF-T (HKUST-1 type), were successfully synthesized using acid-leached EPS as the metal source. Among them, EPS-MOF-T exhibited a high BET surface area of 617.8 m^2^/g and enhanced crystallinity. CO_2_ adsorption measurements demonstrated that EPS-MOF-T achieved a maximum uptake of 1.07 mmol/g at 20 °C, significantly outperforming EPS-MOF-D. The adsorption behavior followed an exothermic physisorption mechanism (Δ*H* ≈ – 22 kJ/mol), supported by isotherm modeling using the Redlich–Peterson equation. In addition to gas adsorption, both MOFs exhibited antibacterial activity against *Escherichia coli*, which is primarily attributed to controlled Cu^2+^ ion release, with ligand-associated reactive oxygen species (ROS) generation acting as a complementary pathway. This work highlights the potential of waste-derived MOFs as multifunctional materials for integrating carbon capture and antimicrobial performance, providing a sustainable approach for electroplating sludge valorization.

## Graphical abstract

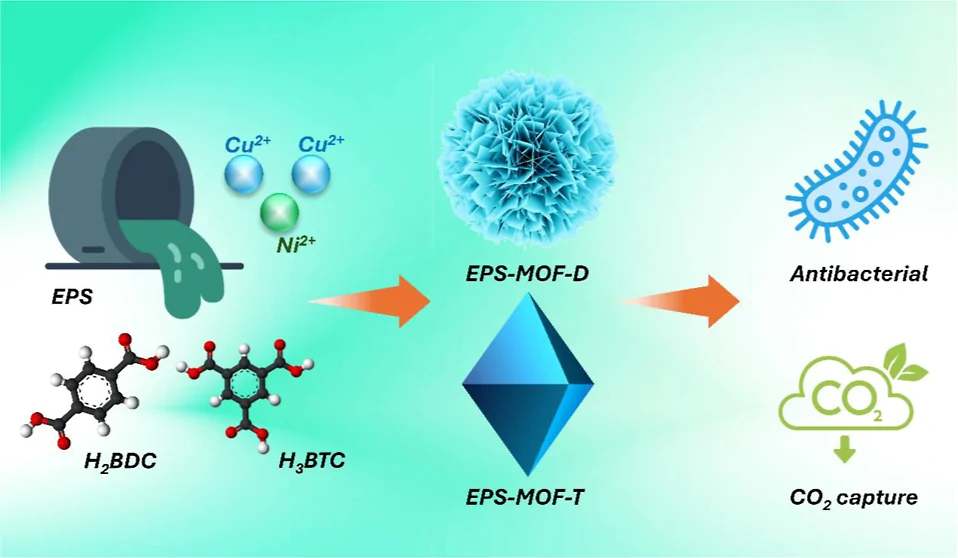

## 1 Introduction

Electroplating sludge (EPS) is a hazardous industrial byproduct generated in substantial quantities from metal finishing and semiconductor packaging processes. Due to its high content of heavy metals such as copper, nickel, and zinc, EPS poses serious environmental threats and regulatory challenges.^1–3^ Traditional disposal methods, including landfilling and solidification, are costly and unsustainable, often failing to meet modern environmental standards.^4–7^ However, the high metal content of EPS also presents an opportunity for resource recovery and valorization within the framework of a circular economy.^8–10^ Metal–organic frameworks (MOFs) are a class of porous crystalline materials composed of metal centers coordinated with organic ligands. Their high surface areas, tunable pore sizes, and diverse functionalities make them attractive for a wide range of applications, including gas adsorption, catalysis, and antimicrobial treatment.^11,12,39^ Most conventional MOF syntheses, however, rely on high-purity metal salts and organic solvents, raising concerns over cost and environmental impact. Recent efforts have turned toward sustainable alternatives, such as the use of waste-derived precursors or biomass-based components for MOF fabrication.^13–15^ Despite this progress, the direct transformation of industrial sludge—particularly EPS—into functional MOF materials remains largely unexplored.

In this work, EPS was utilized as a metal source for the synthesis of MOFs using two common organic linkers: 1,4-benzenedicarboxylic acid (BDC) and 1,3,5-benzenetricarboxylic acid (BTC). The resulting EPS-derived MOFs were systematically characterized and evaluated for two key environmental functions: carbon dioxide (CO_2_) adsorption and antibacterial activity. These dual applications address both climate change mitigation and antimicrobial challenges, particularly in the context of sustainable material development. To the best of our knowledge, this is one of the first studies to demonstrate the dual environmental applications of EPS-derived MOFs. The synthesis approach not only valorizes hazardous sludge waste into high-performance porous materials but also integrates carbon capture and antibacterial functionalities, presenting a viable strategy for sustainable materials development within the circular economy framework.

## 2 Materials and Methods

### 2.1 Reagents and Chemicals

Electroplating sludge (EPS) was collected from ASE Technology Holding Co., Ltd. (Taiwan). Benzene-1,4-dicarboxylic acid (H_2_BDC, 99%) and ethanol (EtOH, 95%) were purchased from Emperor Chemical (Taiwan). Benzene-1,3,5-tricarboxylic acid (H_3_BTC, 98%) was obtained from Thermo Scientific (Taiwan). Dimethylacetamide (DMAC, ≥99.5%) was supplied by Echo Chemical (Taiwan). Hydrochloric acid (HCl, 37%, extra pure reagent) and nitric acid (HNO_3_, 69.0–71.0%, EP grade) were obtained from Union Chemical Works Ltd. (Taiwan). Deionized (DI) water (≥15 MΩ·cm) was used throughout the experiments for solution preparation and washing steps.

### 2.2 Preparation of EPS, EPS-MOF-D, and EPS-MOF-T

The synthesis procedure was adapted from ref 16 with modifications, and the procedure is illustrated in Figure 1a. Prior to synthesis, the collected EPS was dried at 80 °C for 12 h and subsequently calcined at 400 °C in a muffle furnace to obtain thermally treated EPS, which served as the precursor for MOF synthesis. To synthesize EPS-MOF-D, 10 g of the calcined EPS was dispersed in 50 mL of 5 M HCl and stirred for 3 h to leach metal ions. After centrifugation, the supernatant was mixed with a ligand solution containing 12 g of H_2_BDC, 768 mL of DMAC, and 48 mL of ethanol. The mixture was stirred for 30 min and then transferred to a Teflon-lined stainless-steel autoclave for hydrothermal reaction at 140 °C for 24 h. The resulting solid was washed thoroughly with DMAC and ethanol, followed by vacuum drying at 70 °C overnight.

**Figure 1:**
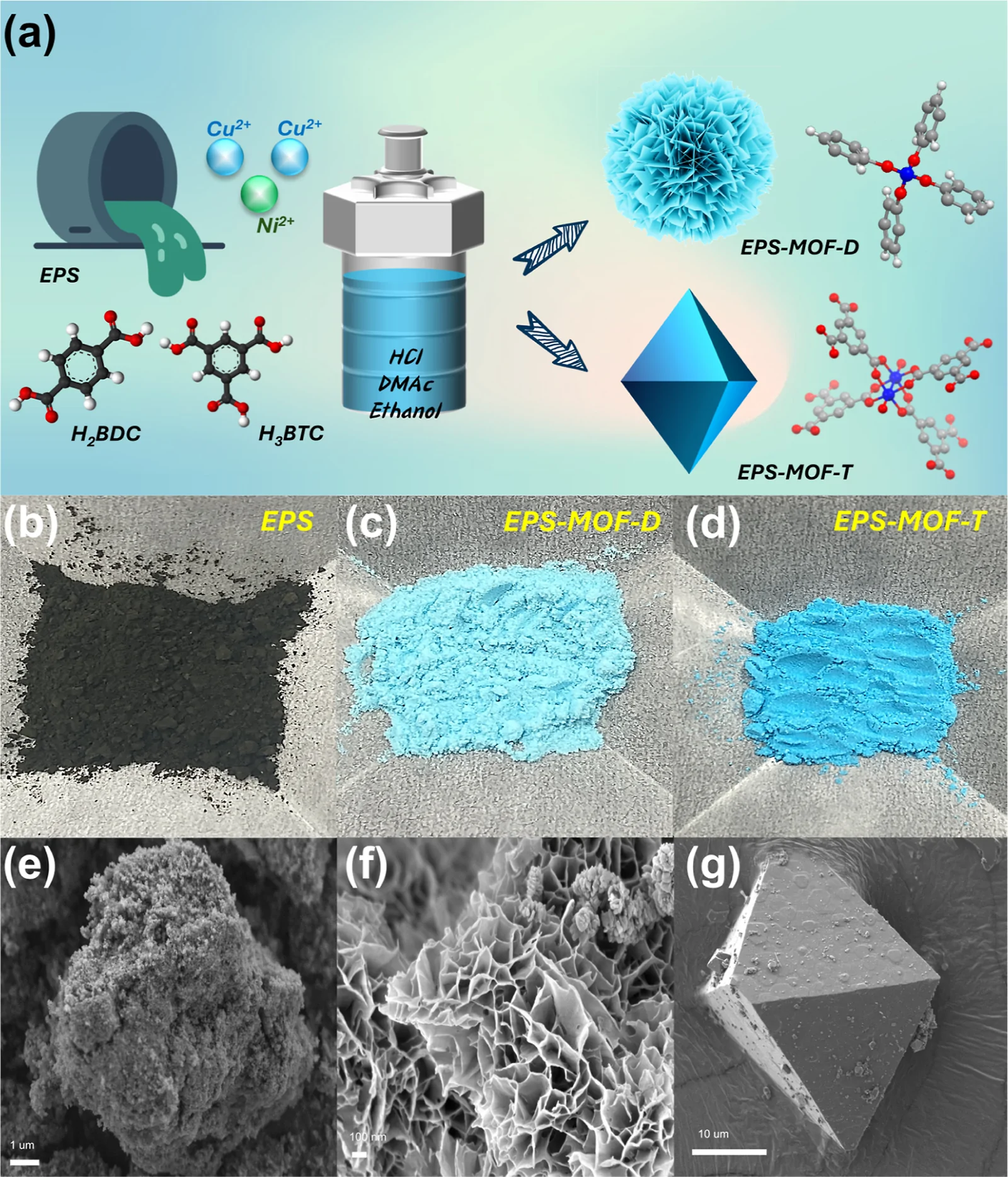
(a) Schematic synthesis of EPS-MOF-D and EPS-MOF-T from electroplating sludge using H_2_BDC and H_3_BTC ligands; photographs of (b) EPS, (c) EPS-MOF-D, and (d) EPS-MOF-T; SEM images of (e) EPS, (f) EPS-MOF-D, and (g) EPS-MOF-T.

For the synthesis of EPS-MOF-T, 10 g of calcined EPS was added to 50 mL of 5 M HNO_3_ and stirred for 3 h. After centrifugation, the obtained metal-containing solution was mixed with a ligand solution composed of 8 g of H_3_BTC, 250 mL of deionized water, and 250 mL of ethanol. The mixture was stirred for 30 min and then subjected to hydrothermal treatment at 120 °C for 12 h. The final product was washed with DMAC and ethanol and vacuum-dried at 80 °C overnight.

### 2.3 Characterization of EPS, EPS-MOF-D, and EPS-MOF-T

In this work, the physicochemical properties of the materials were comprehensively characterized by using a range of advanced analytical techniques. The crystalline phases were identified by using an X-ray diffractometer (XRD, Bruker, USA). The surface morphology and internal structure of the samples were visualized through scanning electron microscopy (SEM, JEOL, Japan) and high-resolution transmission electron microscopy (TEM, JEOL, Japan). The textural parameters, including specific surface area and pore size distribution, were measured using a nitrogen sorption analyzer (NOVA touch, Anton Paar, Austria), while particle size profiles were obtained by using a laser-based particle size analyzer (LA-960, HORIBA, Japan). Thermal behavior was evaluated via thermogravimetric analysis (TGA) using a TGA system (TGA 4000, PerkinElmer, USA). The functional groups of EPS, EPS-MOF-D, and EPS-MOF-T were analyzed through infrared spectroscopy (FT-IR, iS5, Thermo Fisher Scientific, USA), providing insights into their chemical bonding. The electronic states and surface elemental compositions were determined by X-ray photoelectron spectroscopy (XPS, PHI 5000, ULVAC-PHI, Japan). In addition, the concentration of *Escherichia coli* in the microplate during antimicrobial experiments was tracked by ultraviolet–visible spectroscopy (UV–vis, SPARK, Tecan Austria GmbH, Austria).

### 2.4 CO_2_ Adsorption Experiment

A thermogravimetric analyzer was used to evaluate the CO_2_ adsorption behavior of the synthesized EPS-derived MOF materials. Adsorption experiments were conducted at three different temperatures (20 °C, 30 °C, and 40 °C) under varying CO_2_ concentrations (10 vol %, 50 vol %, and 100 vol %) to examine both temperature and concentration effects. To assess gas selectivity, control tests were also carried out using 100% N_2_ under identical conditions. The adsorption procedure involved an initial degassing step, where the sample was heated to 105 °C under a constant flow of nitrogen and held for 30 min to remove physisorbed moisture. Afterward, the system was cooled to the target temperature. Once thermal equilibrium was reached, the purge gas was switched to CO_2_ (at the designated concentration) or to N_2_, and the sample’s weight change was continuously recorded over a period of 120 min. The CO_2_ adsorption capacity was calculated based on the mass difference before and after gas exposure; the detailed formula and calculation method are provided in the Supporting Information.

### 2.5 Antimicrobial Experiment

The antibacterial performance of the samples was assessed using the agar well diffusion method, minimum inhibitory concentration (MIC), and minimum bactericidal concentration (MBC) assays. *Escherichia coli* (*E. coli*) was chosen as a model Gram-negative bacterium. *E. coli* cultures were incubated at 37 °C for 24 h prior to testing. For the agar well diffusion test, a bacterial suspension was adjusted to 0.5 McFarland standard (∼1 × 10^6^ CFU/mL) and uniformly spread on sterile agar plates using a glass spreader. Precompacted EPS-MOF-D and EPS-MOF-T samples were placed onto the agar surface, and the plates were incubated at 37 °C for 24 h. The inhibition zone was measured as the clear area surrounding the sample, indicating complete bacterial growth suppression.

MIC and MBC values were determined using the broth microdilution method in a 96-well plate. Sample stock solutions were prepared in 5% DMSO and serially diluted to final concentrations ranging from 64 to 4096 μg/mL. Each well received 50 μL of diluted sample and 50 μL of bacterial suspension (1 × 10^6^ CFU/mL), followed by incubation at 37 °C for 24 h. The MIC was defined as the lowest concentration that completely inhibited visible bacterial growth. To determine the MBC, 100 μL aliquots from nonturbid wells were plated onto nutrient agar and incubated for another 24 h at 37 °C. The MBC was recorded as the lowest concentration at which no bacterial colonies were observed, indicating complete bactericidal activity.

## 3 Results and Discussion

### 3.1 Characterization of EPS and EPS-Derived MOFs

The transformation of electroplating sludge (EPS) into metal–organic frameworks (MOFs) is illustrated in Figure 1a. Photographs of the materials (Figure 1b–d) demonstrate a clear visual change from the dense, dark EPS powder to light-blue crystalline products, confirming the successful formation of coordination structures. SEM images (Figure 1e–g) reveal substantial morphological differences among the samples. The raw EPS (Figure 1e) exhibited a compact, irregular, and aggregated morphology with no apparent crystalline features. After coordination with H_2_BDC, the resulting EPS-MOF-D (Figure 1f) showed a distinctive nanoflower-like morphology composed of thin, interconnected nanosheets with a wrinkled surface texture, indicating the formation of a porous structure. In contrast, EPS-MOF-T synthesized with H_3_BTC (Figure 1g) displayed well-defined octahedral crystal structures, characteristic of the HKUST-1 type framework.^17,18^ The uniformity and sharp facets of EPS-MOF-T suggest a higher degree of crystallinity and structural ordering compared to EPS-MOF-D. To further evaluate the elemental composition before and after MOF formation, EDS analysis was conducted (Figure S1). The raw EPS (Figure S1a) was found to contain primarily Cu (53.00 wt %) and O (37.53 wt %), with minor Ni content (6.52 wt %), indicating that Cu serves as the dominant coordination center during MOF synthesis. After ligand coordination, the carbon content increased substantially in both EPS-MOF-D (19.19 wt %) and EPS-MOF-T (37.81 wt %), confirming successful incorporation of the organic linkers. Notably, Ni content decreased markedly in EPS-MOF-D (4.18 wt %) and was undetectable in EPS-MOF-T, suggesting preferential coordination of Cu over Ni in the formation of crystalline MOF structures. These results highlight the effective transformation of Cu-rich sludge into high-purity MOF materials and the minimal incorporation of Ni, which may be due to its lower affinity for the selected ligands under the applied hydrothermal conditions.

Further structural insights were obtained from TEM and high-resolution TEM (HR-TEM) analyses, as presented in Figure 2. The raw EPS (Figure 2a) exhibits amorphous aggregated structures, lacking defined crystalline domains. In contrast, EPS-MOF-D (Figure 2b) displays thin nanosheet-like layers consistent with the nanoflower-like morphology observed in SEM, while EPS-MOF-T (Figure 2c) reveals well-faceted octahedral crystals. HR-TEM images (Figures 2d and 2f) show distinct lattice fringes with interplanar spacings of approximately 0.20 and 0.21 nm for EPS-MOF-D and EPS-MOF-T, respectively, confirming their crystalline nature. Additionally, SAED patterns (Figures 2e and 2g) display sharp diffraction rings and spots, further indicating the formation of well-ordered frameworks.

**Figure 2:**
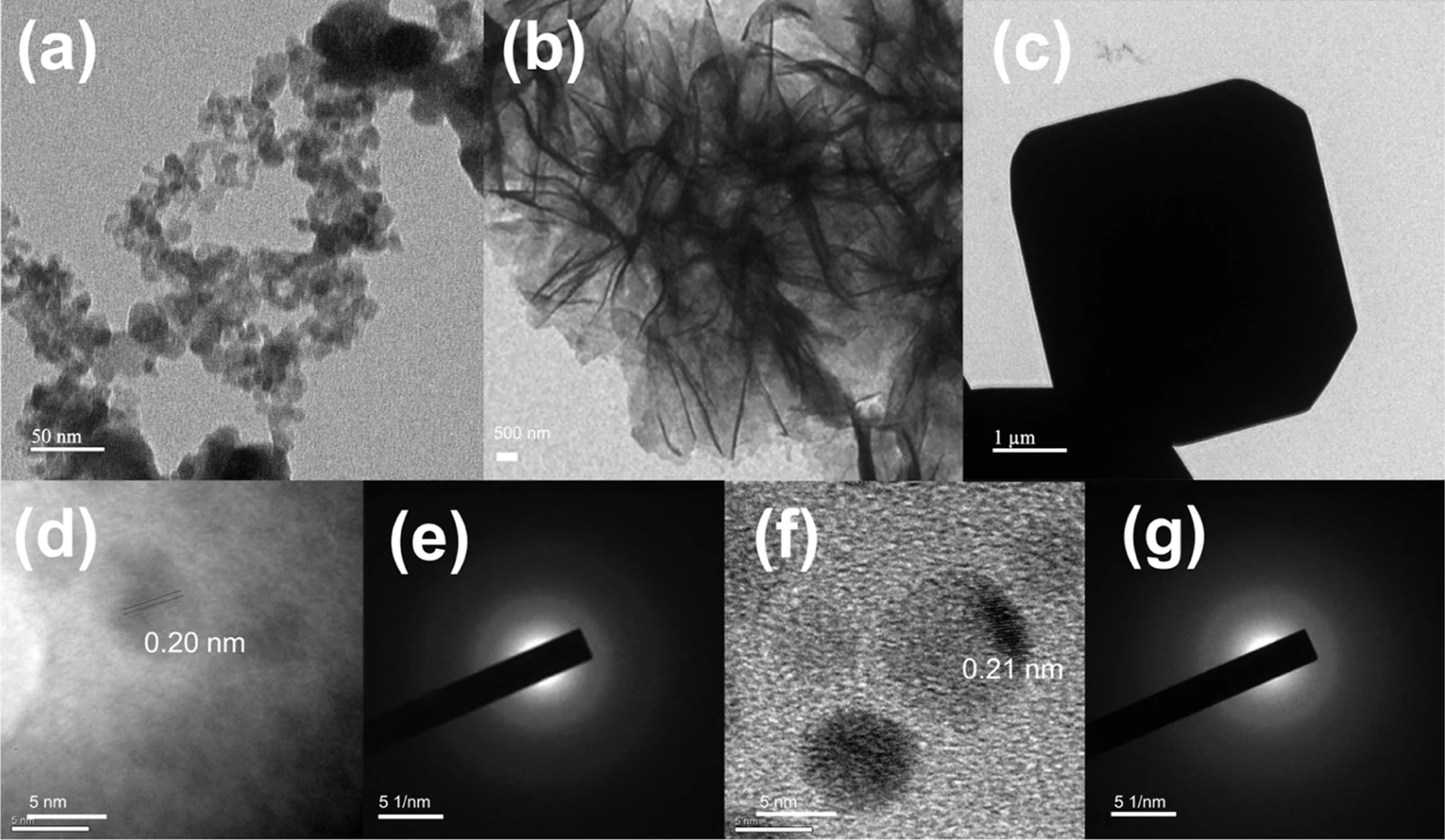
TEM images for the (a) EPS, (b) EPS-MOF-D, and (c) EPS-MOF-T; (d) HR-TEM image and (e) SAED pattern for the EPS-MOF-D; (f) HR-TEM image and (g) SAED pattern for the EPS-MOF-T.

Furthermore, to investigate the structural and physicochemical features of EPS and the synthesized MOFs, FTIR, XRD, TGA, and dynamic light scattering (DLS) analyses were conducted, as shown in Figure 3. In the FTIR spectra (Figure 3a), the raw EPS exhibits a broad – OH stretching band near 3400 cm^–1^ and weak peaks around 600–700 cm^–1^, which can be attributed to Cu–O vibrations. After MOF formation, distinct absorption bands appear near 1600 cm^–1^ and 1400 cm^–1^ in both EPS-MOF-D and EPS-MOF-T, corresponding to the asymmetric and symmetric stretching vibrations of coordinated carboxylate groups (COO^–^), confirming successful metal–ligand coordination. Additional characteristic peaks in EPS-MOF-D (e.g., at 745 and 830 cm^–1^) are related to the C–H bending of the aromatic BDC ligand, while the band at ∼1710 cm^–1^ in EPS-MOF-T indicates the presence of C=O functionalities from BTC.^19,20^ XRD patterns (Figure 3b) show that the EPS precursor lacks long-range crystallinity, as evidenced by broad and weak diffraction signals. In contrast, the synthesized MOFs display sharp and well-defined peaks. EPS-MOF-D shows diffraction peaks assignable to typical Cu-BDC frameworks,^21^ while EPS-MOF-T matches the HKUST-1-type structure,^22^ with prominent reflections at 2θ ≈ 6.7°, 9.5°, and 11.7°, corresponding to the (220), (222), and (400) planes, respectively.

**Figure 3:**
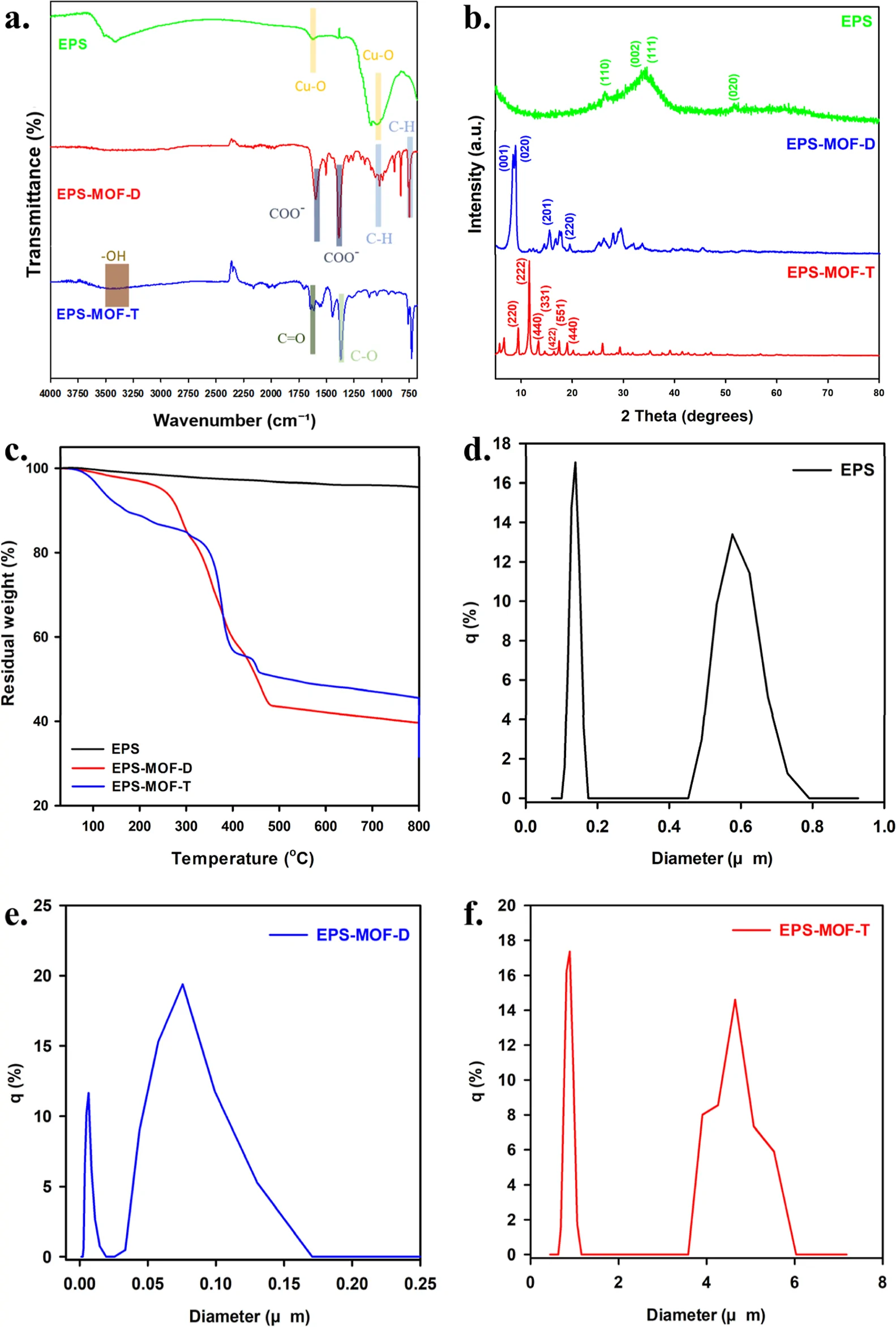
(a) FTIR spectra, (b) XRD patterns, (c) TGA curves, and DLS particle size distribution profile of (d) EPS, (e) EPS-MOF-D, and (f) EPS-MOF-T.

TGA (Figure 3c) reveals a multistage thermal decomposition behavior for the MOF samples. EPS-MOF-D exhibits a major weight loss between 300 and 500 °C due to decomposition of the organic ligands, while EPS-MOF-T shows an earlier onset of degradation at ∼250 °C and a larger overall weight loss, likely due to its higher organic content and porous structure.^23,24^ The raw EPS remains relatively stable, with only minor loss under 200 °C. The DLS results (Figure 3d–f) clearly demonstrate changes in particle size distribution after MOF formation. The raw EPS (Figure 3d) shows a bimodal distribution with peaks at ∼0.2 μm and ∼ 0.6 μm, indicating heterogeneous particle aggregation. Upon coordination with organic linkers, EPS-MOF-D (Figure 3e) displays a narrowed distribution with most particles between 0.01 and 0.15 μm, suggesting effective breakdown of aggregates and formation of finer particles. Similarly, EPS-MOF-T (Figure 3f) exhibits two primary populations centered around ∼1.5 μm and ∼ 4.5 μm, likely reflecting partial agglomeration of HKUST-type crystallites. These size distributions confirm that MOF synthesis alters the morphology and dispersion of the original EPS matrix, providing materials with reduced particle sizes and more defined physical characteristics suitable for adsorption applications.

The porosity and surface textural properties of EPS and the synthesized MOFs were evaluated through N_2_ adsorption–desorption measurements, as shown in Figure 4 and Table S1. The N_2_ sorption isotherms (Figure 4a) of EPS and EPS-MOF-D exhibit typical type IV curves with H3-type hysteresis, indicating the presence of mesopores. In contrast, EPS-MOF-T displays a hybrid isotherm behavior with a steep increase at low relative pressure (P/P_0_ < 0.01), suggesting the presence of micropores, followed by gradual uptake in the mesopore region. The pore size distribution (Figure 4b) shows that EPS and EPS-MOF-D mainly contain mesopores ranging from 4 to 15 nm, while EPS-MOF-T exhibits a narrow distribution concentrated at ∼2 nm. This is further confirmed by the magnified micropore region in Figure 4c, where EPS-MOF-T shows a sharp peak between 1.8 and 2.0 nm, indicating dominant microporosity.

**Figure 4:**
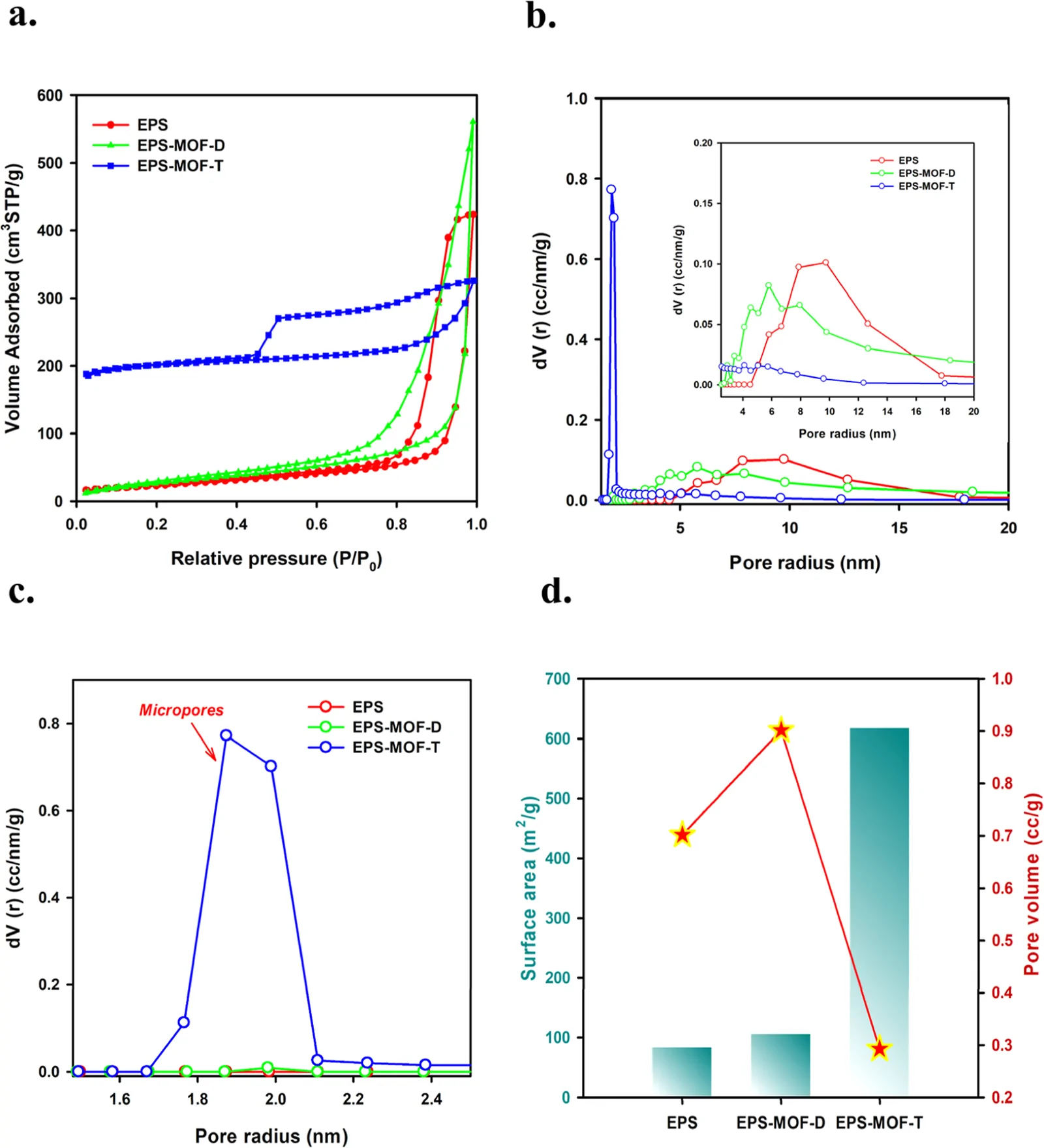
(a) N_2_ adsorption–desorption isotherms, (b) pore size distribution, (c) micropore analysis, and (d) BET surface area and total pore volume of EPS, EPS-MOF-D, and EPS-MOF-T.

Quantitative analysis of the surface area and total pore volume (Figure 4d and Table S1) reveals a substantial difference among the samples. The BET surface area (*S*_BET_) of EPS-MOF-T reaches 617.8 m^2^/g, which is significantly higher than those of EPS (83.4 m^2^/g) and EPS-MOF-D (106.0 m^2^/g). Notably, despite its highest surface area, EPS-MOF-T possesses a lower total pore volume (0.2928 cc/g) compared with EPS-MOF-D (0.9017 cc/g), which is attributed to its predominantly microporous structure. In contrast, the larger pore volume of EPS-MOF-D originates from its mesoporous architecture.

According to gas adsorption theory,^25–27^ materials with high surface area, narrow pore size distribution, and dominant microporosity are more favorable for the capture of small gas molecules such as CO_2_, due to enhanced van der Waals interactions and increased adsorption potential within confined pore environments. In particular, micropores with diameters between 0.5 and 2 nm are considered ideal for CO_2_ adsorption under ambient conditions. Based on this principle, the textural properties of EPS-MOF-T suggest strong potential for superior CO_2_ adsorption performance.

### 3.2 Effect of Temperature on CO_2_ Adsorption Kinetics and Thermodynamics

The CO_2_ adsorption performance of EPS and the synthesized MOFs was investigated under varying temperature and pressure conditions to elucidate their kinetic characteristics, thermodynamic properties, and equilibrium adsorption behavior. As shown in Figure 5a, at 30 °C, EPS-MOF-T exhibited the highest adsorption capacity, reaching 0.74 mmol/g, significantly surpassing that of EPS-MOF-D (0.24 mmol/g) and raw EPS (0.18 mmol/g). This superior uptake performance is attributed to EPS-MOF-T’s microporous architecture and high surface area, as discussed in Section 3.1. The effect of temperature was further evaluated to understand the adsorption mechanism. As shown in Figure 5b,c, the CO_2_ uptake decreased with increasing temperature for both MOFs, indicating an exothermic adsorption process.^28,29^ For EPS-MOF-D, the capacity dropped from 0.34 mmol/g at 20 °C to 0.19 mmol/g at 40 °C, while EPS-MOF-T showed a more significant decline, from 1.07 mmol/g to 0.60 mmol/g over the same range. These trends are typical of physisorption, where lower temperatures enhance van der Waals interactions between CO_2_ and the adsorbent surface.

**Figure 5:**
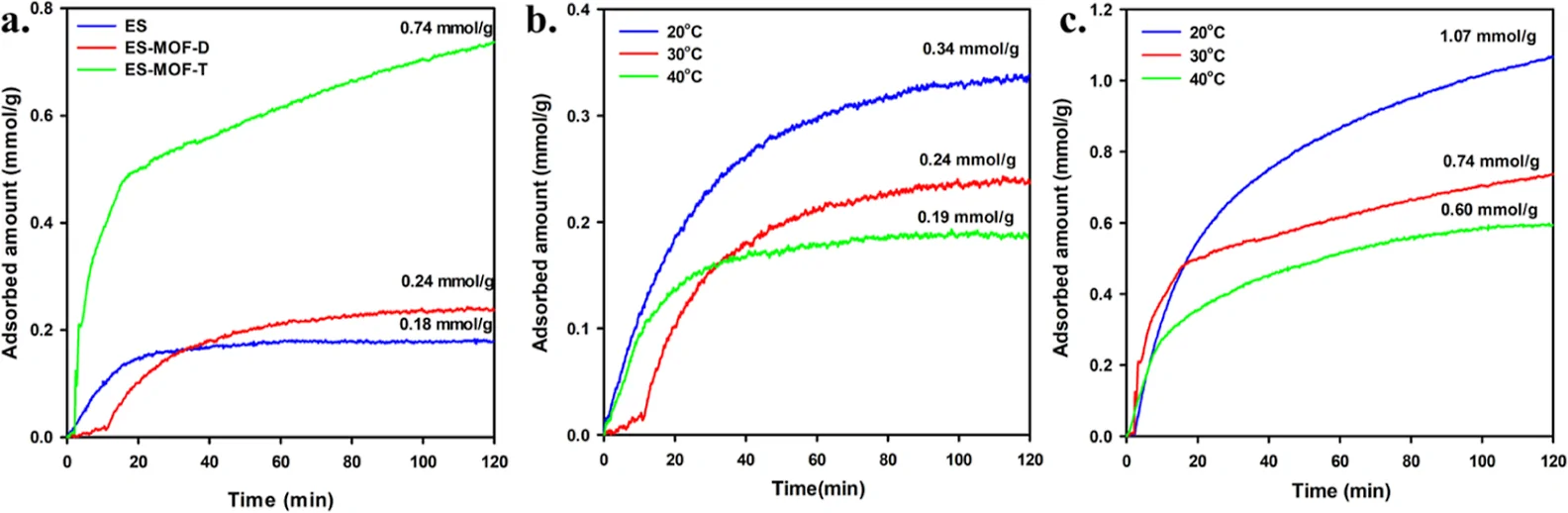
Time-dependent CO_2_ adsorption of (a) EPS, EPS-MOF-D, and EPS-MOF-T at 30 °C; (b) EPS-MOF-D and (c) EPS-MOF-T under different temperatures (20, 30, and 40 °C), measured in mmol/g over 120 min ([CO_2_] = 10 vol %, materials = 10 mg, Total flow = 20 mL/min).

Although EPS-MOF-T exhibited a CO_2_ adsorption capacity of approximately 1.07 mmol/g at 20 °C, this value is lower than those reported for benchmark HKUST-1 materials. For example, recent studies reported CO_2_ adsorption capacities of 5.34 and 5.84 mmol/g for HKUST-1 under their respective measurement conditions.^30,31^ This difference may be partly attributed to the use of electroplating sludge as the metal precursor in the present study. Unlike MOFs synthesized from high-purity metal salts, sludge-derived precursors may contain residual impurities and secondary metal species that can interfere with framework formation and reduce the accessibility of adsorption sites. Previous studies have also demonstrated that changes in accessible pore volume can substantially influence the CO_2_ uptake of HKUST-1.^31^ Therefore, residual species in EPS-MOF-T may partially obstruct the pore structure and limit CO_2_ diffusion and adsorption within the framework.

The thermodynamic parameters calculated from van’t Hoff analysis are summarized in Table S2. Both MOFs exhibited negative enthalpy changes (Δ*H* ≈ – 22 kJ/mol), reaffirming the exothermic nature of adsorption. Notably, EPS-MOF-T displayed more favorable Gibbs free energy (Δ*G*) values across all temperatures (e.g., 0.64 kJ/mol at 30 °C) compared to EPS-MOF-D (3.52 kJ/mol), indicating a more spontaneous adsorption process. In addition, EPS-MOF-T had a less negative entropy change (Δ*S* = – 75.13 J/mol·K) than EPS-MOF-D (−84.99 J/mol·K), suggesting a more accessible and less ordered adsorption environment, likely due to its open pore structure and uniform distribution of active sites.

To complement the temperature-dependent analysis, pressure-dependent CO_2_ adsorption isotherms were examined and fitted with Langmuir, Freundlich, and Redlich–Peterson models, as shown in Figure 6. For both EPS-MOF-D (Figure 6a) and EPS-MOF-T (Figure 6b), the Redlich–Peterson model yielded the best fit (R^2^ = 0.9976 and 1.0000, respectively), indicating a hybrid adsorption mechanism involving both monolayer and heterogeneous surface interactions. The corresponding fitting parameters and goodness-of-fit values for the three isotherm models are summarized in Table S3. The superior maximum adsorption capacity of EPS-MOF-T (∼3.2 mmol/g) under elevated CO_2_ pressure further highlights the advantages of its microporous framework for gas uptake. These results reinforce the thermodynamic observations and confirm the strong physical affinity of EPS-MOF-T toward CO_2_ due to its higher surface area, favorable pore environment, and strong yet reversible physisorption interaction with CO_2_ molecules.

**Figure 6:**
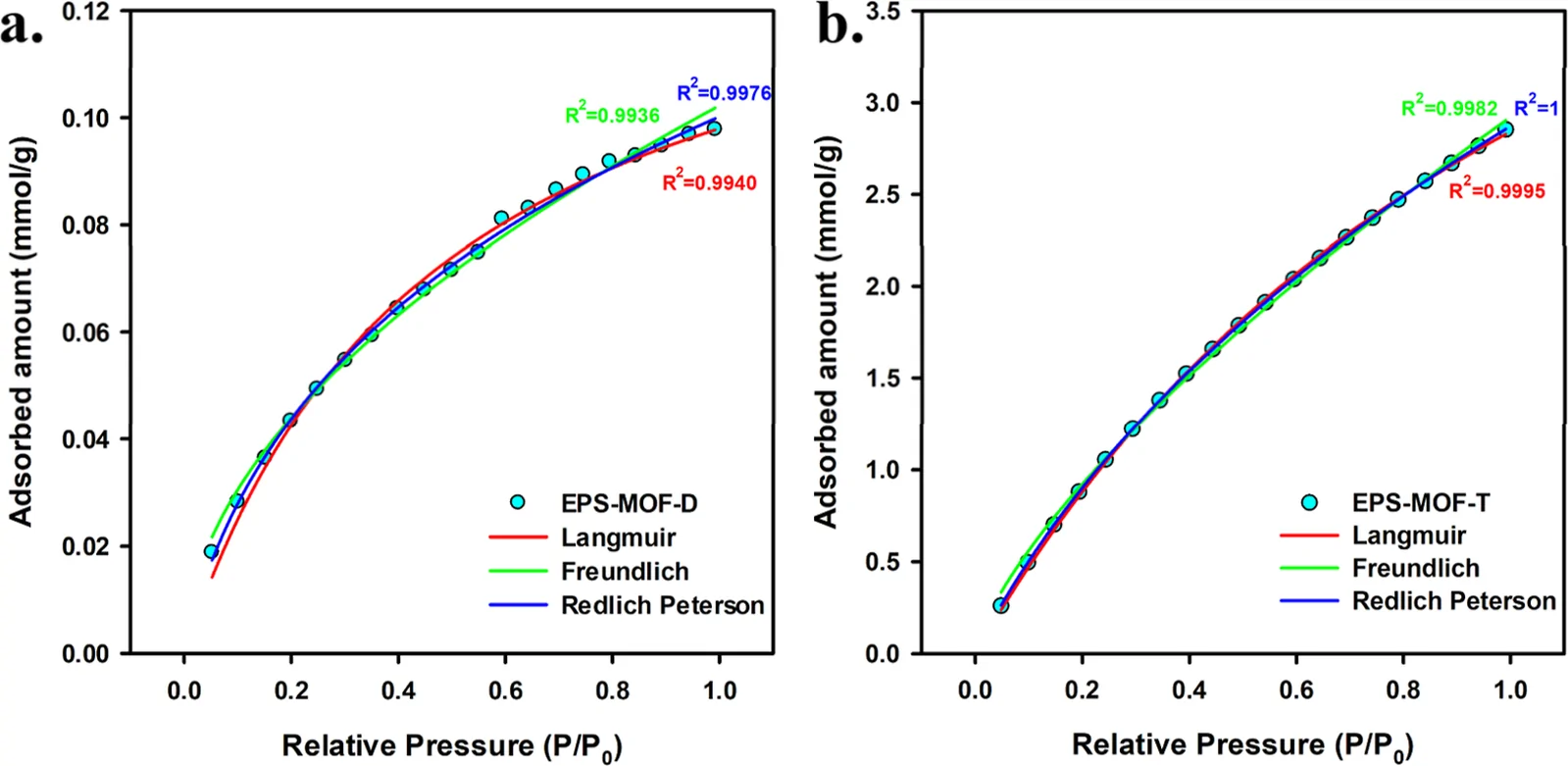
CO_2_ adsorption isotherms at 20 °C for (a) EPS-MOF-D and (b) EPS-MOF-T, fitted with Langmuir, Freundlich, and Redlich–Peterson models.

### 3.3 Effect of CO_2_ Concentration and Reusability and Mechanistic Analysis

The adsorption performance of EPS-derived MOFs was further evaluated under varying CO_2_ concentrations and multiple adsorption–desorption cycles to assess their practical applicability and adsorption stability. As shown in Figures 7a and 7b, increasing the CO_2_ concentration from 10 to 100 vol % led to a pronounced increase in adsorption capacity for both EPS-MOF-D and EPS-MOF-T. For EPS-MOF-D, the uptake increased from 0.24 to 0.45 mmol/g, while EPS-MOF-T showed a much steeper rise from 0.74 to 2.87 mmol/g. This concentration-dependent enhancement is characteristic of physisorption, where a higher partial pressure of CO_2_ increases the driving force for adsorption. In contrast, both materials exhibited negligible adsorption under pure N_2_ flow, confirming their high CO_2_/N_2_ selectivity. This indicates that the adsorbents interact more strongly with CO_2_ due to its quadrupole moment and polarizability, while N_2_, being less polarizable, is poorly retained in the MOF frameworks.^32,33^ Such selective adsorption is desirable for CO_2_ separation in mixed gas systems.

**Figure 7:**
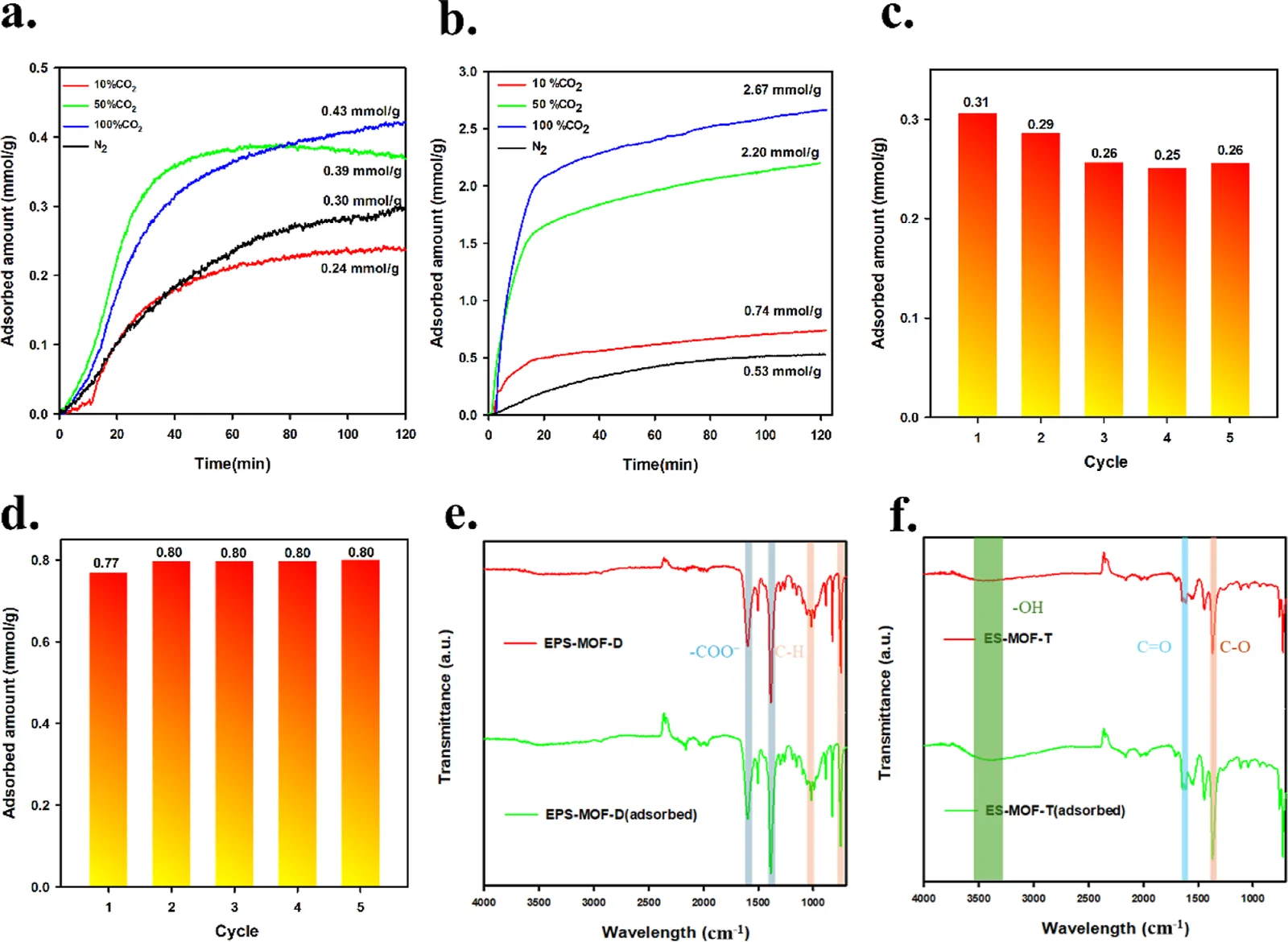
Effect of different CO_2_ concentrations on (a) EPS-MOF-D and (b) EPS-MOF-T; recyclability of CO_2_ adsorption using (c) EPS-MOF-D and (d) EPS-MOF-T; comparison of FTIR spectra before and after CO_2_ adsorption of (e) EPS-MOF-D and (f) EPS-MOF-T.

Notably, EPS-MOF-T not only exhibited higher uptake but also demonstrated greater responsiveness to changes in the CO_2_ concentration compared to EPS-MOF-D. This suggests a larger number of accessible micropores and stronger confinement effects, which facilitate the preferential CO_2_ capture. The sharp difference between 10 and 100 vol % CO_2_ adsorption further highlights the dynamic adsorption capacity of EPS-MOF-T and its potential for pressure-swing or concentration-swing adsorption processes. Reusability tests (Figures 7c and 7d) further demonstrate the stability of the materials. EPS-MOF-T maintained an over 0.80 mmol/g adsorption capacity after five cycles, while EPS-MOF-D showed a gradual decline from 0.31 to 0.28 mmol/g. This highlights the structural robustness and reversible adsorption characteristics of the EPS-MOF-T.

Furthermore, in order to investigate the adsorption mechanism, FTIR and XPS analyses were conducted, and the results, as illustrated in Figures 7e and 7f, show that both EPS-MOF-D and EPS-MOF-T exhibited noticeable changes in their FTIR spectra after CO_2_ adsorption. In particular, the carboxylate stretching bands (O=C–O) around 1580–1400 cm^–1^ showed reduced intensity, and the broad – OH vibration near 3400 cm^–1^ also decreased, especially in EPS-MOF-T. These observations suggest that CO_2_ molecules may weakly associate with oxygen-containing functional groups, such as – COO^–^ and – OH, through hydrogen bonding or dipole interactions. The absence of new absorption bands further supports that no strong covalent bonds were formed during adsorption.

XPS analysis provided additional insight into the chemical state of surface elements before and after CO_2_ exposure (Figures 8 and S2). The Cu 2p spectra for both EPS-MOF-D and EPS-MOF-T maintained characteristic peaks at ∼ 934.5 eV (Cu 2p_3_/_2_) and ∼ 954.4 eV (Cu 2p_1_/_2_) with satellite features, confirming that Cu remained in the +2 oxidation state. No binding energy shift or peak broadening was observed, indicating that Cu sites were not chemically involved in CO_2_ binding. The C 1s spectra revealed a slight decrease in the O = C–O component (∼288.5 eV) after adsorption, along with minor attenuation of the C–O peak (∼286.2 eV). More evidently, the O 1s spectra displayed decreased intensity for both the C=O (∼531.6 eV) and C–O (∼533.0 eV) peaks, particularly in EPS-MOF-D. These reductions imply that CO_2_ adsorption may partially shield or occupy oxygen-rich surface environments, leading to decreased photoelectron emission from those sites. However, the lack of new peaks (e.g., carbonate species) and unchanged binding energies further confirm that adsorption proceeds via physisorption rather than chemisorption. The full survey XPS spectra (Figure S2) confirmed the chemical stability of both MOFs, with no signs of degradation or new element incorporation after CO_2_ exposure. Combined with the minimal spectral shifts and excellent adsorption reversibility, these results indicate that CO_2_ uptake is primarily governed by weak physical interactions, such as van der Waals forces and electrostatic attractions within micropores. The slight decrease in oxygen-related signals likely reflects transient site occupation or local electron redistribution during adsorption.

**Figure 8:**
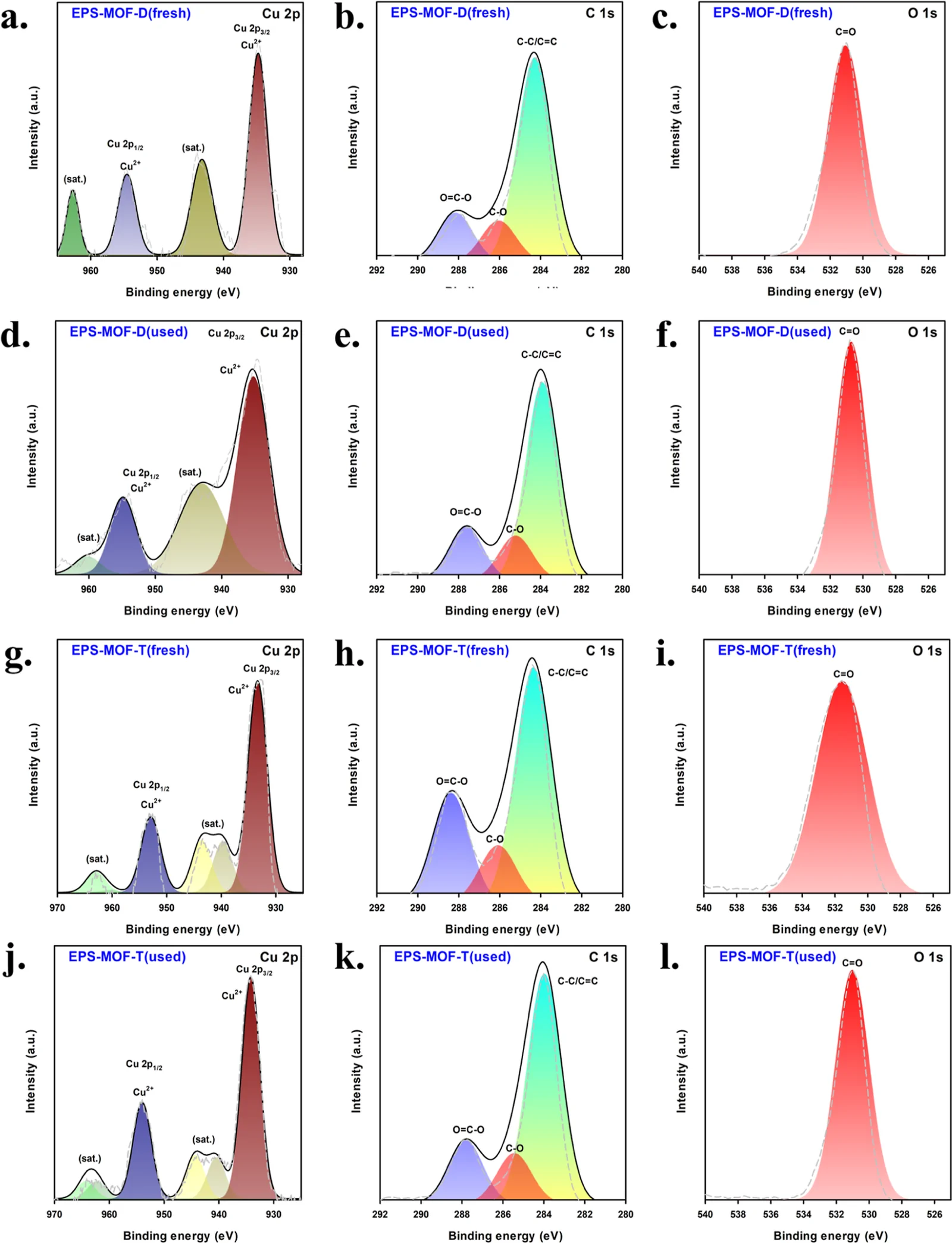
XPS spectra of EPS-MOF-D and EPS-MOF-T before and after CO_2_ adsorption: (a,d,g,j) Cu 2p spectra, (b,e,h,k) C 1s spectra, and (c,f,i,l) O 1s spectra. (a–c) fresh EPS-MOF-D; (d–f) used EPS-MOF-D; (g–i) fresh EPS-MOF-T; (j–l) used EPS-MOF-T.

### 3.4 Antibacterial Activity

The antibacterial performance of EPS-MOF-D and EPS-MOF-T was evaluated through minimum inhibitory concentration (MIC), minimum bactericidal concentration (MBC), and inhibition zone assays to investigate their potential for antimicrobial applications. As shown in Figure 9a,b, both EPS-MOF-D and EPS-MOF-T exhibited concentration-dependent antibacterial activity against the Gram-negative bacterium *E. coli*, the tested bacterial strain. For EPS-MOF-D (Figure 9a), bacterial growth was significantly suppressed at concentrations of 1024 μg/mL, and EPS-MOF-T (Figure 9b) achieved complete growth inhibition at a similar concentration to EPS-MOF-D.

**Figure 9:**
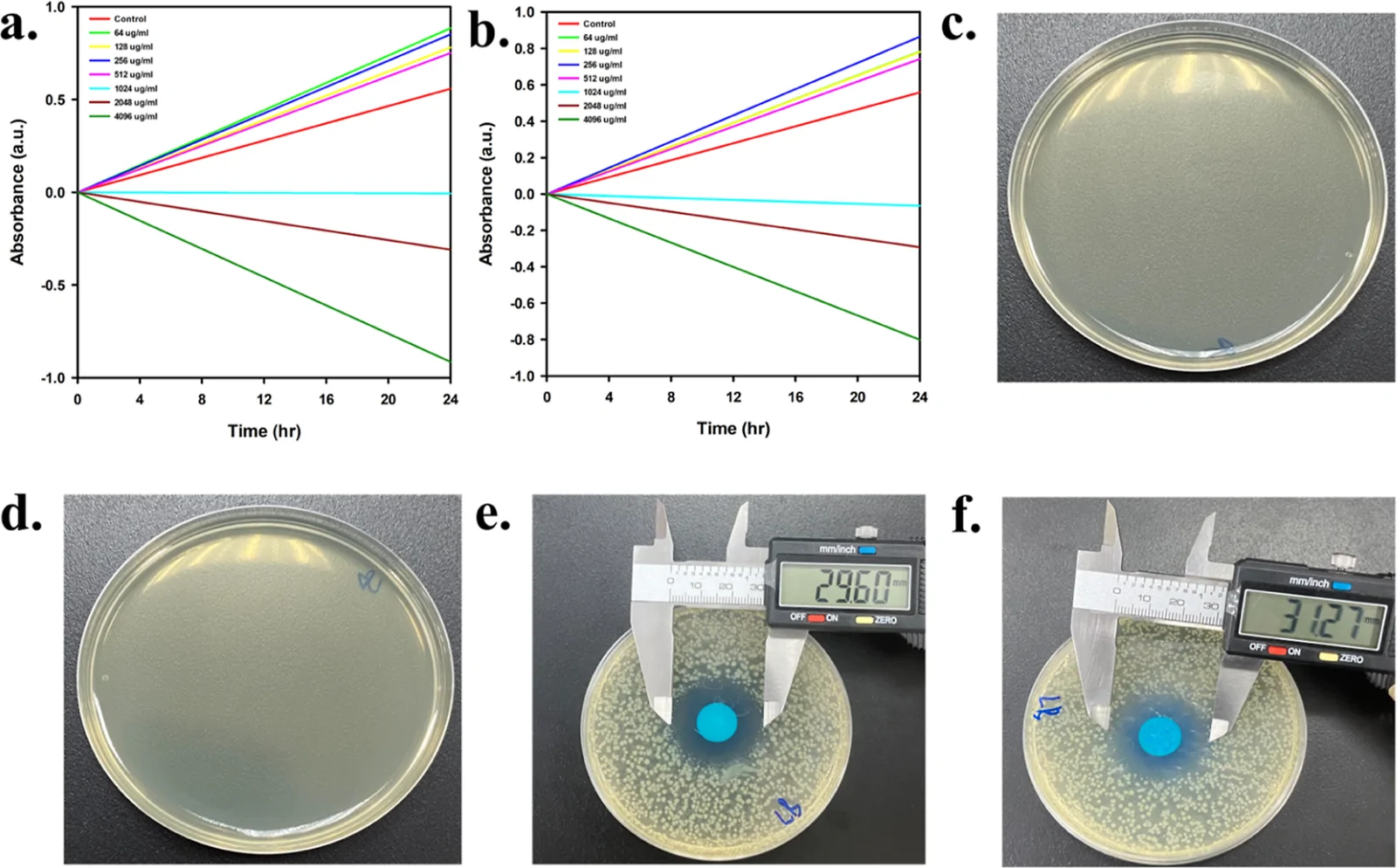
MIC experiment of (a) EPS-MOF-D and (b) EPS-MOF-T; MBC experiment of (c) EPS-MOF-D and (d) EPS-MOF-T; inhibition zone diameter of (e) EPS-MOF-D and (f) EPS-MOF-T.

The MBC results (Figure 9c,d) showed no visible bacterial colonies for the concentrations tested in 2048 μg/mL, confirming bactericidal effects for both MOFs. The disk diffusion results (Figure 9e,f) further supported these findings, showing that both EPS-MOF-D and EPS-MOF-T demonstrated visible inhibition effects against bacterial growth over 24 h. Initially, no inhibition zones were observed at 0 h for all groups, including the blank paper disk (Figure 10a). However, after 24 h of incubation, clear inhibition zones appeared around the MOF samples (Figure 10b,c), with EPS-MOF-T exhibiting a larger inhibition zone (31.27 mm) compared to EPS-MOF-D (29.60 mm), indicating stronger antimicrobial efficacy. A summary of the quantitative antibacterial results—including MIC, MBC, and inhibition zone diameters—is presented in Table 1, which further confirms the comparable minimum inhibitory and bactericidal concentrations of both materials while highlighting the slightly enhanced zone of inhibition exhibited by EPS-MOF-T.

**Figure 10:**
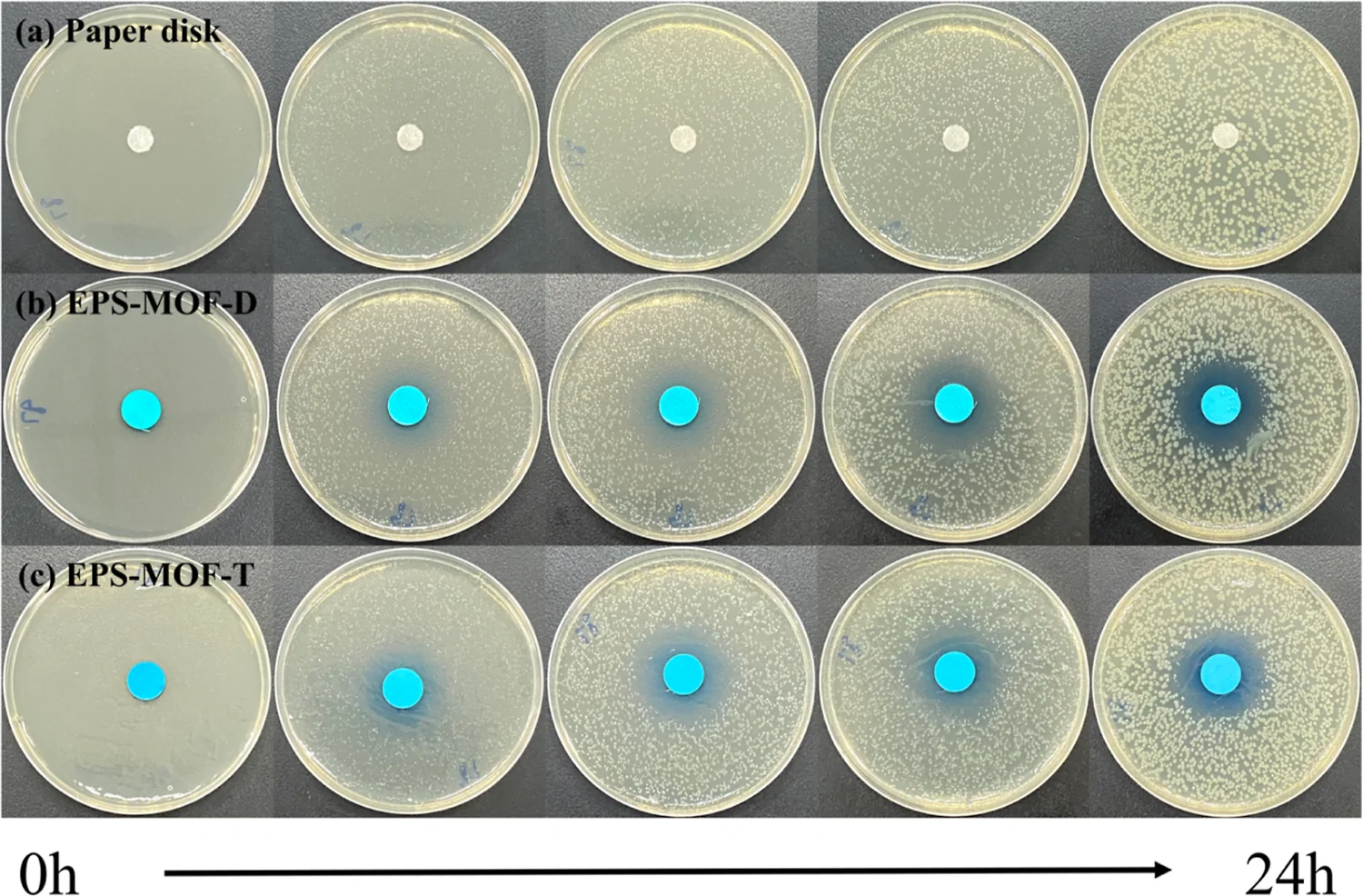
Antibacterial process of (a) paper disk, (b) EPS-MOF-D, and (c) EPS-MOF-T.

**Table 1:** Antibacterial Activity Data of EPS-MOF-D and EPS-MOF-T

materials	MIC (ug/mL)	MBC (ug/mL)	inhibition zone (mm)
EPS-MOF-D	1024	2048	29.60
EPS-MOF-T	1024	2048	31.27

The antibacterial mechanism of the synthesized MOFs is supposed to involve direct interaction with bacteria, in which the antibacterial effect of MOFs relies primarily on their structural and physicochemical properties.^34^ The difference in the clear inhibition zones can be correlated with their distinct structural characteristics. The enhanced antimicrobial performance of EPS-MOF-T can be attributed to its well-defined octahedral crystal morphology and higher degree of crystallinity, which likely facilitates more effective bacterial contact. The antibacterial action is supposed to involve disruption of bacterial cell membranes;^35,36^ nevertheless, more microscopic investigations are necessary to confirm these mechanisms.^37^

Moreover, the organic ligands in MOFs not only serve as structural linkers coordinating with metal centers to create a potent framework, but they also actively contribute to the antibacterial activity. Notably, the ligands used—BDC and BTC—can directly promote the generation of reactive oxygen species (ROS) through spontaneous or metal–ligand-mediated electron transfer mechanisms, thereby enhancing the antibacterial efficacy of the material.^38^

As shown in Table S4, most recent sludge-derived MOFs have primarily been developed for individual applications, such as contaminant adsorption, catalytic degradation, or moisture capture.^16,39–42^ In contrast, the present study demonstrates the integration of CO_2_ adsorption and antibacterial activity within EPS-derived MOFs. In particular, EPS-MOF-T combines a relatively high BET surface area (617.8 m^2^/g), CO_2_ adsorption capability, and antibacterial performance against *E. coli*. These findings extend the application scope of EPS-derived MOFs beyond conventional single-purpose environmental applications and demonstrate their potential as multifunctional materials for sustainable waste valorization.

Therefore, this study is among the first to showcase the dual environmental benefits of EPS-derived MOFs, transforming hazardous sludge waste into high-performance porous materials while simultaneously incorporating carbon capture and antibacterial properties, offering a promising pathway for sustainable material development within a circular economy framework.

## 4 Conclusion

In this study, a sustainable and waste-derived approach was developed to convert electroplating sludge (EPS) into bifunctional MOF materials for environmental applications. Two EPS-derived MOFs—EPS-MOF-D (Cu-BDC type) and EPS-MOF-T (HKUST-1 type)—were synthesized via ligand-assisted solvothermal coordination using acid-leached EPS as the metal source. Among them, EPS-MOF-T exhibited a higher surface area (617.8 m^2^/g), more defined crystallinity, and smaller particle size, as confirmed by XRD, BET, and DLS analyses. CO_2_ adsorption evaluations revealed that EPS-MOF-T achieved a maximum uptake of 1.07 mmol/g at 20 °C, significantly outperforming EPS-MOF-D (0.34 mmol/g). The adsorption process followed exothermic physisorption behavior, as indicated by negative enthalpy changes (Δ*H* ≈ – 22 kJ/mol) and pressure-dependent isotherm data best fitted to the Redlich–Peterson model (R^2^ = 1.000). These results suggest that CO_2_ capture occurs via weak van der Waals interactions and pore filling within microporous domains, facilitated by open metal sites and high pore accessibility. Additionally, both MOFs demonstrated antibacterial activity against *E. coli*, with EPS-MOF-T showing superior performance (MIC = 1024 μg/mL; inhibition zone = 31.3 mm), attributed to enhanced metal ion exposure and surface interaction. The integration of gas capture and microbial inhibition within a single EPS-derived framework highlights a promising waste-to-functional-material strategy that supports a circular economy and sustainable pollution control.

## Supplementary Material



## Data Availability

Data will be made available on request.

## Notes

CO_2_ adsorption capacity calculation; textural properties of EPS and EPS-derived MOFs; thermodynamic parameters; Langmuir, Freundlich, and Redlich–Peterson isotherm fitting parameters; comparison with reported sludge-derived MOFs; EDS analysis; and XPS survey spectra of fresh and used EPS-derived MOFs (PDF)
