# Supplementary material for: Sustainable Synthesis of Electroplating Sludge-Derived MOFs for CO2 Adsorption and Antibacterial Applications
Source: ACS Omega. 2026 Sep 28;11(40):60785–96. doi: 10.1021/acsomega.6c03377 (PMC13670284; doi:10.1021/acsomega.6c03377)
Supplement: Supplementary file 1 [file ao6c03377_si_001.pdf]

Supplementary Information

For

Sustainable Synthesis of Electroplating Sludge-Derived MOFs for CO_2 Adsorption and Antibacterial Applications

Jia-Yin Lin[abc], Si-Han Yu[b], Yun-Ju Lai[a], Chih-Ying Wang[ac], Nai-Wen Tsao[d], Bussaba Pinchaipat[e*]*

[a] Graduate Program in Semiconductor and Green Technology, Academy of Circular Economy, National Chung Hsing University, Taichung 402, Taiwan

[b] Graduate Program in Industrial and Smart Technology, Academy of Circular Economy, National Chung Hsing University, Taichung 402, Taiwan

[c] Innovation and Development Center of Sustainable Agriculture, National Chung Hsing University, Taichung 402, Taiwan

[d] Department of Wood-Based Materials and Design, National Chiayi University, Chiayi 600, Taiwan

[e] Department of Basic Science and Physical Education, Kasetsart University, Bangkok 10900, Thailand

*Corresponding Authors. E-mail addresses: joylin7@dragon.nchu.edu.tw (*Jia-Yin Lin*); bussaba.pin@ku.th (*Bussaba Pinchaipat)*

S1. Determination of CO_2 Adsorption Capacity (Gravimetric Method)

The CO_2 adsorption capacity of the material was calculated based on the difference in weight before and after adsorption, using the following equation:

$$\frac{(\mathrm{Wmax}-\mathrm{Wo})}{\mathrm{Wo}} \times \frac{1}{\mathrm{Mole}(co_2)} \times 1000 \left(\frac{\mathrm{mmol}}{\mathrm{g}}\right) \qquad (1)$$

- W_{max}: weight of the adsorbent (mg) after reaching saturation at time t_s
- W_0: initial weight of the adsorbent (mg)
- $Mole_{(CO2)}$: molar mass of CO_2 (44 g/mol)

Table S1. Textural properties of EPS and EPS-derived MOFs (EPS-MOF-D and EPS-MOF-T).

**Sample**	**S_{BET} (m^2/g)**	**V_t (cc/g)**
EPS	83.4	0.7018
EPS-MOF-D	106.08	0.9017
EPS-MOF-T	617.8	0.2928

Table S2. Thermodynamic analysis of EPS-MOF-D and EPS-MOF-T

**Sample**	**T(°C)**	**ΔH (kJ/mol)**	**ΔS (J/mol·K)**	**ΔG (kJ/mol)**
	20			2.67
ES-MOF-D	30	-22.4	-84.99	3.52
	40			4.37
	20			-0.11
ES-MOF-T	30	-22.13	-75.13	0.64
	40			1.39

Table S3. Fitting parameters of the Langmuir, Freundlich, and Redlich–Peterson models for CO_2 adsorption on EPS-MOF-D and EPS-MOF-T at 20 °C.

Model	Parameter	EPS-MOF-D	EPS-MOF-T
Langmuir	q_m	0.14514	6.40677
	K_l	2.07806	0.79741
	Adj. R^2	0.99402	0.99947
Freundlich	K_F	0.10226	2.92188
	1/n	0.52488	0.71585
	Adj. R^2	0.99364	0.99817
Redlich–Peterson	K_RP	0.49631	6.2007
	a_RP	3.9534	1.15977
	β	0.70611	0.70358
	Adj. R^2	0.99763	1.00000

Table S4. Comparison of recent studies on sludge-derived MOFs and their environmental applications.

Waste source	MOF/material	BET surface area (m^2/g)	Application / performance	Key feature	Ref.
**Electroplating sludge**	**EPS-MOF-D / EPS-MOF-T**	**106.1 / 617.8**	**CO_2 adsorption; 1.07 mmol/g at 20 °C for EPS-MOF-T; antibacterial activity**	**Integration of CO_2 capture and antibacterial functionality in EPS-derived MOFs**	**This work**
Electroplating sludge	ES-MOF-BDC / ES-MOF-BTC	106.036 / 316.493	Fenton-like dye degradation; 95.76% Congo Red removal	Upcycling Cu-rich electroplating sludge into nanoflower-structured Cu-MOF catalysts	[2]
Iron-containing sludge + white sludge	SW-MOF-C	179.1	PMS activation for BPA degradation; kobs = 0.58 min^{-1}	Dual-sludge-derived MOF carbon catalyst	[3]
Semiconductor electroplating sludge	E-MOFs-D / E-MOFs-T	66.10 / 412.16	Water vapor adsorption; maximum uptake of 11.98 mmol/g at 25 °C and 90% RH	Reusable EPS-derived MOF desiccant with life-cycle assessment	[4]
Ni-containing electroplating sludge + waste PET	Ni-MOF	168	Cu(II) adsorption; maximum adsorption capacity of 193.1 mg/g at 328 K	Dual-waste valorization using sludge-derived Ni and PET-derived ligand	[5]

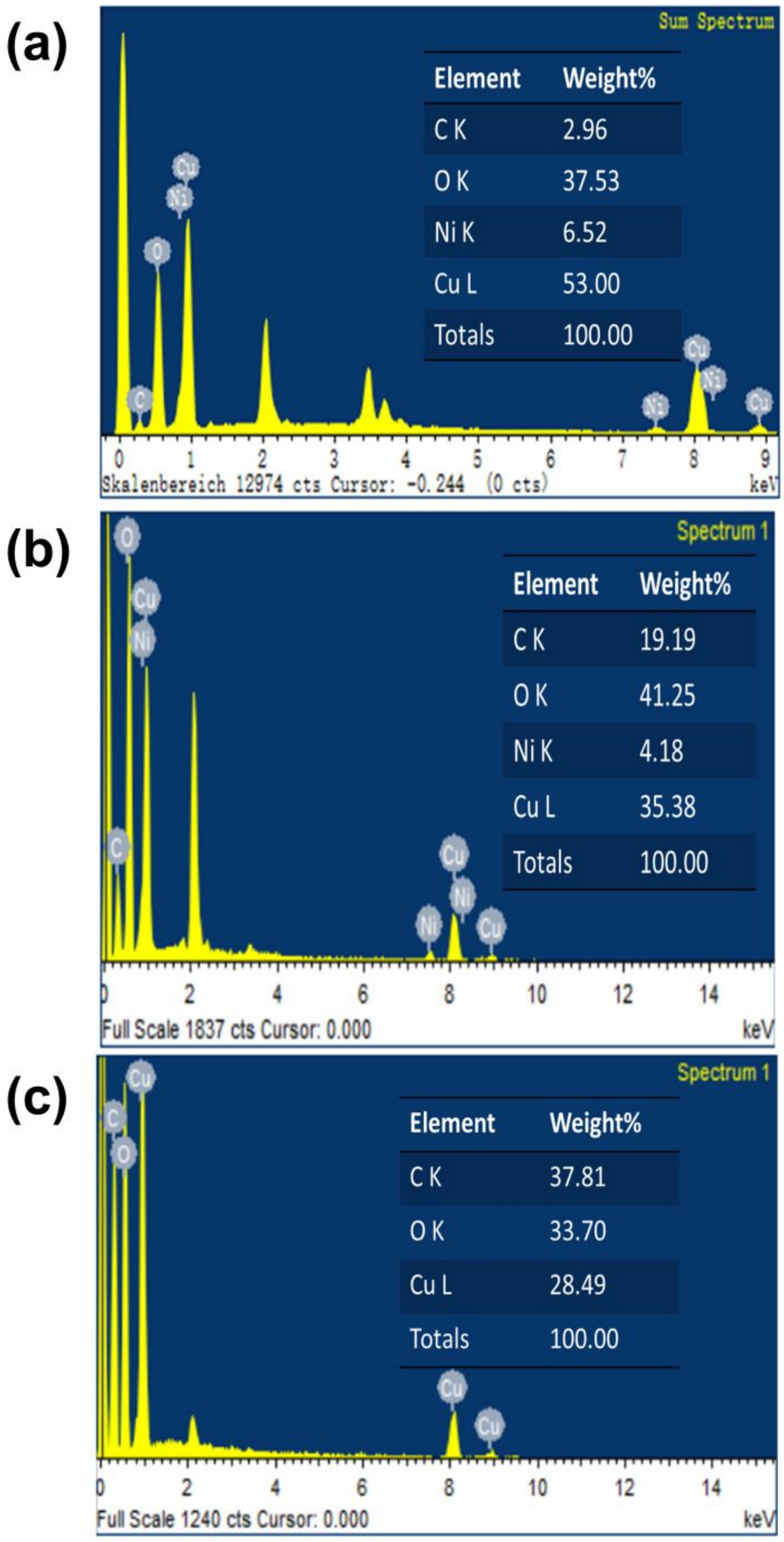

Fig. S1. EDS analysis of (a) raw electroplating sludge (EPS), (b) EPS-MOF-D, and (c) EPS-MOF-T.

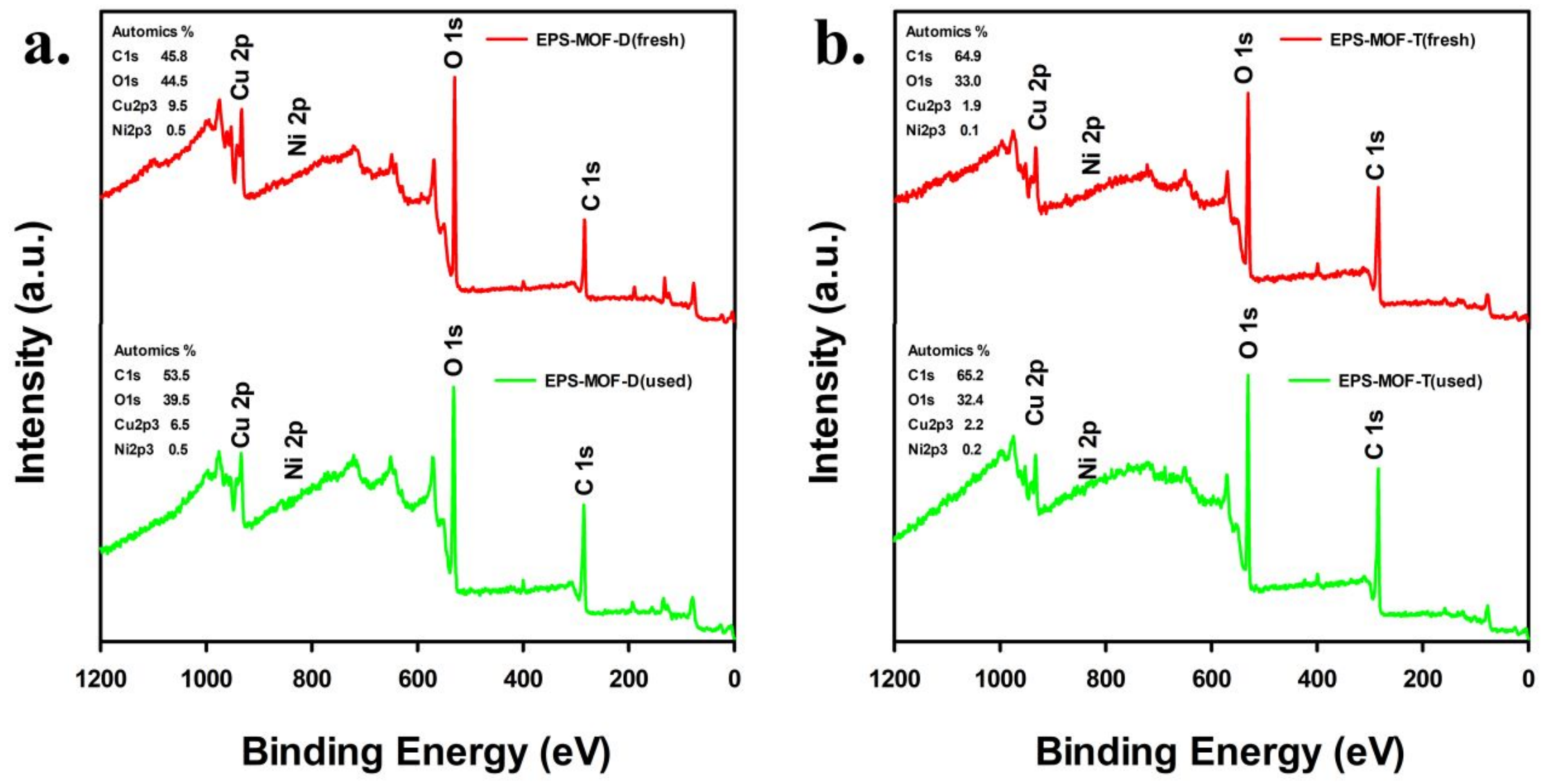

Fig. S2. XPS analysis of full survey spectra of (a) fresh and used EPS-MOF-D, (b) fresh and used EPS-MOF-T

Reference

1. Teng, R., et al., *Synthesis of mesoporous MIL-100 (Fe) from acid mine drainage sludge for norfloxacin removal: Industrial sludge high value utilization, adsorbent performance and contaminant removal mechanisms.* Colloids and Surfaces A: Physicochemical and Engineering Aspects, 2024. **684**: p. 133032.
2. Lin, J.-Y., et al., *Upcycling electroplating sludge into nanoflower-structured Cu-MOFs for efficient Fenton-like degradation of organic dyes.* Journal of Water Process Engineering, 2025. **75**: p. 107983.
3. Yao, Y., et al., *Metamorphoses of industrial waste sludge via metal organic frameworks into efficient Fenton-like catalysts.* Chemical Engineering Journal, 2025. **519**: p. 165620.
4. Lin, J.-Y., et al., *Eco-friendly upcycling of semiconductor sludge into MOFs for sustainable dehumidification: Performance evaluation and life cycle assessment.* Separation and Purification Technology, 2026: p. 138872.
5. Jiang, Y., et al., *Dual-waste valorization toward sustainable Ni-MOF synthesis for selective Cu (Ⅱ) adsorption from wastewater.* Journal of Environmental Chemical Engineering, 2026: p. 123241.
